# Antibiotic Resistance of *Helicobacter pylori* Strains Isolated From Pediatric Patients in Southwest China

**DOI:** 10.3389/fmicb.2020.621791

**Published:** 2021-01-26

**Authors:** Juan Li, Jianjun Deng, Zhiling Wang, Hong Li, Chaomin Wan

**Affiliations:** ^1^Department of Pediatrics, West China Second University Hospital, Sichuan University, Chengdu, China; ^2^Department of Infection Control, West China Second University Hospital, Sichuan University, Chengdu, China; ^3^Key Laboratory of Birth Defects and Related Diseases of Women and Children (Sichuan University), Ministry of Education, Chengdu, China; ^4^West China Marshall Research Center for Infectious Diseases, Center of Infectious Diseases, West China Hospital, Sichuan University, Chengdu, China

**Keywords:** *Helicobacter pylori*, antimicrobial susceptibility testing, pediatric population, empiric therapy, rifampicin

## Abstract

The number of antibiotics that are appropriate for *Helicobacter pylori* eradication in children is limited. Profiling regional or population-specific antibiotic resistance is essential in guiding the *H. pylori* eradication treatment in children. The aim of this study was to evaluate the antibiotic resistance in *H. pylori* strains isolated from children and adolescents in Southwest China. Gastric biopsies from 157 pediatric patients with or without previous *H. pylori* eradication treatment were collected for *H. pylori* culture. Susceptibility to amoxicillin (AML), clarithromycin (CLR), metronidazole (MTZ), levofloxacin (LEV), tetracycline (TET), furazolidone (FZD), and rifampicin (RIF) was determined by E-test or a disk diffusion assay. A total of 87 patients from three ethnic groups (Han/Tibetan/Yi) were *H. pylori* culture positive (55.4%). The overall resistance rates were 55.2% for CLR, 71.3% for MTZ, 60.9% for RIF, and 18.4% for LEV. No isolate was found to be resistant to AML, TET, and FZD. Among the 53 treatment-naïve pediatric patients, primary resistance rates to clarithromycin, metronidazole, levofloxacin, and rifampicin were 45.3, 73.6, 15.1, and 60.4%, respectively. Among the 34 treatment-experienced patients, secondary resistance rates to clarithromycin, metronidazole, levofloxacin, and rifampicin were 70.6, 67.6, 23.5, and 61.8%, respectively. Isolates exhibiting simultaneous resistance to clarithromycin and metronidazole were 28.3 and 52.9% among the treatment-naïve and treatment-experienced patients, respectively. In conclusion, among pediatric patients in Southwest China, resistance rates were high for clarithromycin, metronidazole, levofloxacin, and rifampicin, whereas nil resistance was found to amoxicillin, tetracycline, and furazolidone. Our data suggest that the standard clarithromycin-based triple therapy should be abandoned as empiric therapy, whereas the bismuth quadruple therapy (bismuth/PPI/amoxicillin/tetracycline) would be suitable as first-line empiric treatment regimen for this pediatric population. Tetracycline and furazolidone may be considered for treating refractory *H. pylori* infections in adolescent patients.

## Introduction

*Helicobacter pylori* infection is a common bacterial infectious disease, the major cause of chronic active gastritis, peptic ulcers, and one of the most important risk factors for gastric cancer and mucosa-associated lymphoid tissue (MALT) lymphoma (Malfertheiner et al., 2017; Liu et al., 2018). It has been recommended that all adult patients with positive test of active *H. pylori* infection should be offered with *H. pylori* eradication therapy (Sugano et al., 2015; Chey et al., 2017; Malfertheiner et al., 2017). However, in comparison with adults, children, and adolescents with *H. pylori* infection rarely develop serious diseases or related symptoms (Jones et al., 2017; Liu et al., 2018). In addition, as *H. pylori* is usually acquired in childhood, the reinfection rate in children after successful *H. pylori* eradication is higher than in adults (Jones et al., 2017; Liu et al., 2018). Thus, the “test and treat” strategy is not suitable for *H. pylori* infection in children under 14years of age (Liu et al., 2018). However, for children with peptic ulcer diseases, “test and treat” for *H. pylori* infection is required (Jones et al., 2017; Liu et al., 2018).

The number of antibiotics that are appropriate for *H. pylori* eradication in children is limited. The current recommended first-line *H. pylori* eradication regimens in children are mainly triple therapies consisting of a proton pump inhibitor (PPI) plus two antibiotics chosen from amoxicillin (AML), clarithromycin (CLR), and metronidazole (MTZ) for a duration of 14days (Xu et al., 2015; Jones et al., 2017). The tetracycline (TET), furazolidone (FZD), and levofloxacin (LEV) that are appropriate for use in adults with *H. pylori* infection are relatively contradicted in Children due to potential side effects. LEV is usually contraindicated in children younger than 18years due to potential severe side effects including tendon rupture. However, the use of TET can be considered in children older than 8years (Xu et al., 2015; Jones et al., 2017). With the availability of FZD in China, the use of FZD may be considered in >14-year-old children with refractory *H. pylori* infections.^1^

Given the limited number of antibiotics that are appropriate for *H. pylori* eradication in children, and the worldwide increase of antibiotic resistance, the recent ESPGHAN/NASPGHAN guidelines have recommended antimicrobial susceptibility testing to guide the *H. pylori* eradication treatment in children (Jones et al., 2017). However, due to the fastidious culture of *H. pylori* and the lack of an easy and cost-effective testing method, antimicrobial susceptibility testing for *H. pylori* is almost universally unavailable in medical centers (Montes and Pérez-Trallero, 2017; Li et al., 2019; Tang et al., 2020a,b). Under such circumstances, profiling regional or population-specific antibiotic resistance patterns is of great importance in guiding the development of effective empiric treatment regimens. As the *H. pylori* antibiotic resistance profiles among children and adolescents in Southwest China is lacking, the aim of this study was to evaluate *H. pylori* strains isolated from this population for antibiotic resistance to CLR, MTZ, LEV, AML, TET, FZD, and rifampicin (RIF).

## Materials and Methods

### Patients

Patients with severe upper gastrointestinal symptoms referred to West China Second University Hospital from January 2019 to December 2019 for gastrointestinal endoscopy were considered for this study. Patients older than 18years, with severe systemic illness, use of PPI or antibiotics in the last month; and previous gastric surgery were excluded. Informed consent was obtained from the pediatric patients’ parents or guardians. This study was approved by the ethics committee of West China Second University Hospital, Sichuan University (HXEY2019011). A total of 157 pediatric patients from three different ethnic groups (Han, Tibetan, and Yi) were enrolled in this study.

### *Helicobacter pylori* Culture and Antimicrobial Susceptibility Testing

Gastric biopsy samples collected from the gastric antrum and/or corpus of the 157 pediatric patients were placed in sterile vials containing Brain Heart Infusion (BHI) medium supplemented with 20% glycerol and 5% new-born calf serum (NCS) and transferred on ice to the *H. pylori* laboratory at West China-Marshall Research Center for Infectious Diseases. The samples were stored at −80°C until for culture of *H. pylori*. For *H. pylori* culture, frozen gastric samples were thawed, homogenized, and inoculated onto both non-selective commercial Columbia Blood Agar (CBA) plates (Auto bio, China) and selective CBA plates supplemented with Dent antibiotics (Oxoid, United Kingdom). Inoculated plates were incubated at 37°C under microaerobic conditions (5% O_2_, 10% CO_2_, and 85% N_2_) generated using the Anoxomat Mark-II system (Mart Microbiology B.V., the Netherlands). Colonies displaying typical *H. pylori* morphology were confirmed by Gram-staining, positive urease-, catalase-, and oxidase-tests.

The isolated *H. pylori* strains were tested for susceptibility to CLR, MTZ, LEV, AML, TET, and RIF using the Epsilometer test strips (Liofilchem s.r.l, Italy), whereas susceptibility to FZD was determined using agar dilution method as previously described (Su et al., 2013).

According to the European Committee on Antimicrobial Susceptibility Testing (EUCAST), *H. pylori* resistance to CLR, MTZ, LEV, TET, and RIF were defined as the minimal inhibitory concentration (MIC) >0.5mg/L, >8mg/L, >1mg/L, >1mg/L, and >1mg/L, respectively (Kahlmeter et al., 2006). Base on a very recent clinical study (Graham et al., 2020), resistance to AML was defined as MIC >0.25mg/L. Resistance to FZD was defined as MIC >2mg/L (Kahlmeter et al., 2006; Su et al., 2013). The *H. pylori* strain NCTC 11637 was used for quality control.

### Statistics

Data were statistically analyzed using SPSS version 24.0 (IBM Corporation, Armonk, NY, United States). Chi-square test (*χ*^2^) or Fisher’s exact test was used to investigate factors associated with antibiotic resistance among the pediatric patients. Differences were considered statistically significant with a value of *p* < 0.05.

## Results

Between January 2019 and December 2019, 157 children and adolescents who underwent gastrointestinal endoscopy were included in this study to have their gastric biopsies collected for *H. pylori* culture. A total of 87 patients were found to be *H. pylori* positive (55.4%), 34 were girls and 53 were boys with a mean age of 10.9 ± 2.8years (age range, 5–17years). Fifty-three strains (60.9%, 53/87) were isolated from treatment-naïve patients and the remaining 34 (39.1%, 34/87) strains were isolated from treatment-experienced patients. The main endoscopic findings among the 87 *H. pylori* culture-positive patients were gastritis/duodenitis in 68 (78.2%), followed by duodenal ulcer in 13 (14.9%), gastric ulcer in three (3.4%), and other diagnosis in three (3.4%; Table 1).

**Table 1:** Patient demographics and clinical characteristics.

Variables	Patients (*n* = 87)
**Gender**	
Male/female	53/34
**Race/ethnics**	
Han	59
Tibetan	22
Yi	6
Mean age (years)	10.9 ± 2.8
**Endoscopy findings**	
Gastritis/duodenitis	68
Duodenal ulcer	13
Gastric ulcer	3
Other	3

**Figure 1:**
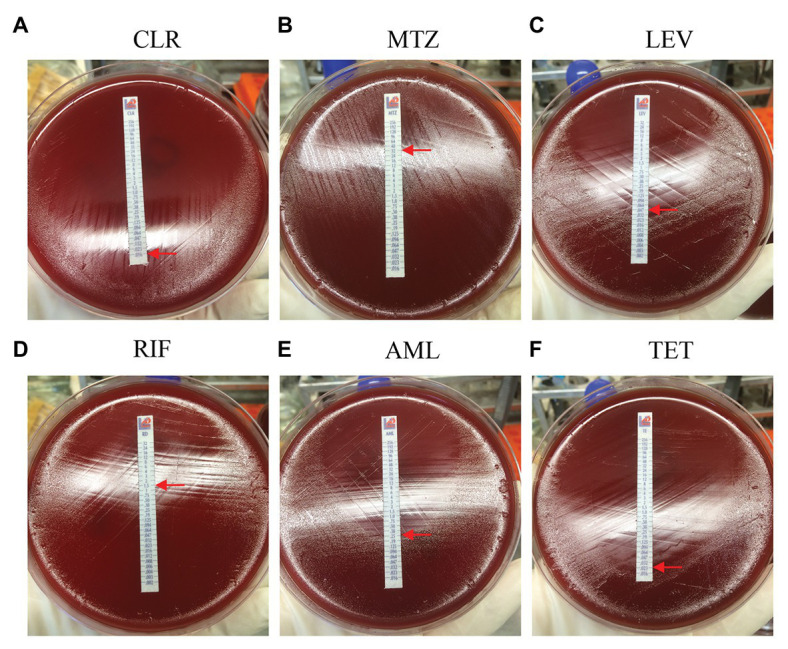
Antibiotic resistance profiles determined by E-test. The minimal inhibitory concentration (MIC) value (the elliptical inhibition zone edge intersecting with the MIC scale on the E-test strip) is indicated by a red arrow. **(A)** CLR, clarithromycin; **(B)** MTZ, metronidazole; **(C)** LEV, levofloxacin; **(D)** RIF, rifampicin; **(E)** AML, amoxicillin; and **(F)** TET, tetracycline.

**Figure 2:**
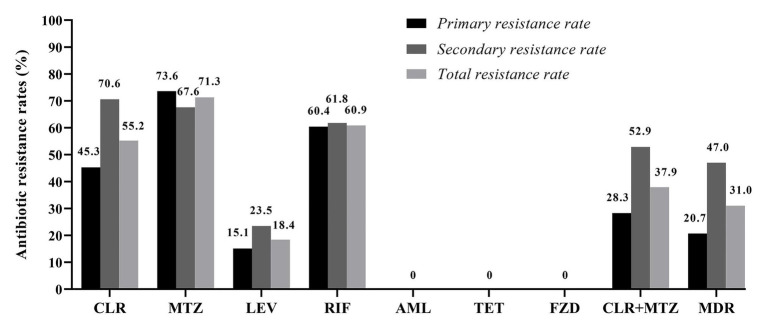
Antibiotic resistance rates among the 87 isolated pediatric strains. CLR, clarithromycin; MTZ, metronidazole; LEV, levofloxacin; RIF, rifampicin; AML, amoxicillin; TET, tetracycline; FZD, furazolidone; and MDR, multidrug resistance. MDR refers strains exhibiting resistance to three or more of the tested antibiotics.

Among the 87 pediatric *H. pylori* strains with resistance profiled using the Epsilometer test strips (Figure 1), the overall antibiotic resistance rates were 55.2% (48/87) for CLR, 71.3% (62/87) for MTZ, 60.9% (53/87) for RIF, and 18.4% (16/87) for LEV. No isolate was found to be resistant to AML, TET, and FZD (Figure 2). Of the 53 strains isolated from patients without previous treatment, resistance rates to CLR, MTZ, LEV, and RIF were 45.3% (24/53), 73.6% (39/53), 15.1% (8/53), and 60.4% (32/53), respectively. Of these 53 strains, 3 (5.7%) were susceptible to all the tested seven antibiotics, 12 were mono-resistant (22.6%), 27 (50.9%) were dual resistant, and the remaining 11 strains were resistant to three or more antibiotics, thus giving an MDR rate of 20.7% (Table 2).

**Table 2:** Antibiotic resistance patterns among the 87 isolated pediatric strains.

Susceptibility testing results	Treatment-naïve (*n* = 53)		Treatment-experienced (*n* = 34)		Overall (*n* = 87)	
	*n*	Resistance (%)	*n*	Resistance (%)	*n*	Resistance (%)
Mono resistance	12	22.6	7	20.6	19	21.8
CLR	5	9.4	1	2.9	6	6.9
MTZ	5	9.4	2	5.9	7	8.0
RIF	2	3.8	3	8.8	5	5.7
LEV	0	0	1	2.9	1	1.1
Dual resistance	27	50.9	10	29.4	37	42.5
CLR + MTZ	5	9.4	4	11.8	9	10.3
CLR + RIF	4	7.5	3	8.8	7	8.0
LEV + MTZ	2	3.8	1	2.9	3	3.4
RIF + MTZ	16	30.2	2	5.9	18	20.7
Triple resistance	7	13.2	15	44.1	22	25.3
CLR + MTZ + RIF	5	9.4	10	29.4	15	17.2
LEV + MTZ + RIF	1	1.9	0	0	1	1.1
CLR + LEV + RIF	0	0	2	5.9	2	2.3
CLR + MTZ + LEV	1	1.9	3	8.8	4	4.6
Quadruple resistance	4	7.5	1	2.9	5	5.7
CLR + MTZ + LEV + RIF	4	7.5	1	2.9	5	5.7

CLR, clarithromycin; MTZ, metronidazole; LEV, levofloxacin; and RIF, rifampicin.

Among the 34 strains isolated from patients with prior *H. pylori* treatment, resistance rates to CLR, MTZ, LEV, and RIF were 70.6% (24/34), 67.6% (23/34), 23.5% (8/34), and 61.8% (21/34), respectively (Figure 2). Of these 34 strains, only one strain (2.9%) was susceptible to all the seven antibiotics, seven strains (20.6%) were mono-resistant, 10 strains (29.4%) were dual resistant, and the remaining 16 strains were MDR (47.0%; Figure 2; Table 2). Of note, the total number of strains exhibiting simultaneous resistance to CLR and MTZ (including the dual and MDR strains) were 15 (28.3%) from the 53 treatment-naïve patients, and 18 (52.9%) from the 34 *H. pylori* treatment-experienced patients (Table 2).

Clarithromycin resistance rate in 5–11-year-old-group was comparable to that of the 12–17-year-old group (57.4 and 52.5%, respectively; Table 3). No significant difference was found in resistance rate to MTZ, LEV, and RIF between the two age groups, either. Among the six strains isolated from Yi ethnic group (all were treatment naïve), one strain (16.7%) was resistant to CLR, whereas five of them (83.3%) were resistant to both MTZ and RIF. However, no significant difference in antibiotic resistance rate was observed among the three different ethnic groups. There was no difference between male and female patients in resistance rates to all the seven antibiotics tested in this study. The 34 strains isolated from patients with prior *H. pylori* treatment were more likely to be resistant to CLR than the 53 strains isolated from *H. pylori* treatment naïve patients (70.6 vs. 45.3%, *p* = 0.02). However, no significant difference was observed between treatment-experienced and treatment-naïve patients in resistant rates to MTZ, LEV, and RIF (Table 3).

**Table 3:** Factors associated with *Helicobacter pylori* resistance to CLR, MTZ, LEV, and RIF in pediatric patients.

	*n*	CLR resistance	*p*	MTZ resistance	*p*	LEV resistance	*p*	RIF resistance	*p*
**Age**									
5–11	47	27 (57.4%)	0.98	35 (74.5%)	0.47	12 (25.5%)	0.06	27 (57.4%)	0.47
12–17	40	21 (52.5%)		27 (67.5%)		4 (10.0%)		26 (65.0%)	
**Race/ethnics**									
Han	59	36 (61.0%)	0.10	45 (76.3%)	0.15	12 (20.3%)	0.25	35 (59.3%)	0.58
Tibetan	22	11 (50.0%)		12 (54.5%)		2 (9.1%)		13 (59.1%)	
Yi	6	1 (16.7%)		5 (83.3%)		2 (33.3%)		5 (83.3%)	
**Gender**									
male	53	25 (47.2%)	0.06	38 (71.7%)	0.91	9 (17.0%)	0.67	33 (62.3%)	0.75
female	34	23 (67.6%)		24 (70.6%)		7 (20.6%)		20 (58.8%)	
**Prior treatment**									
No	53	24 (45.3%)	0.02	39 (73.6%)	0.55	8 (15.1%)	0.32	32 (60.4%)	0.90
Yes	34	24 (70.6%)		23 (67.6%)		8 (23.5%)		21 (61.8%)	

CLR, clarithromycin; MTZ, metronidazole; LEV, levofloxacin; and RIF, rifampicin.

**Figure 3:**
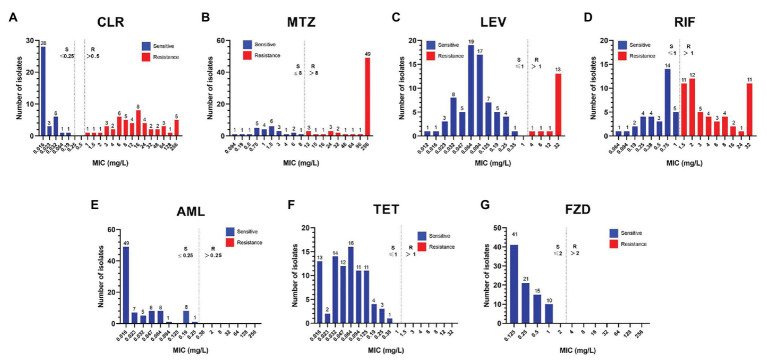
Distribution of the MICs against seven antibiotics among the 87 isolated pediatric strains. The resistance cut-off value for each antibiotic is indicated by a dotted line. **(A)** CLR, clarithromycin; **(B)** MTZ, metronidazole; **(C)** LEV, levofloxacin; **(D)** RIF, rifampicin; **(E)** AML, amoxicillin; **(F)** TET, tetracycline; and **(G)** FZD, furazolidone.

The distribution of the MIC values against seven antibiotics in 87 pediatric *H. pylori* strains was shown in Figure 3. The MIC values against CLR ranged widely: ≤0.25mg/L (*n* = 39), 1–8 (*n* = 19), 12–48 (*n* = 20), and 64–256 (*n* = 9). Among the 62 MTZ-resistant strains, 79.0% (49/62) had high level resistance with MIC ≥256mg/L. All the 71 strains susceptible to LEV had a low MIC value in the range of 0.012–0.35mg/L, while among the 16 LEV-resistant strains, 81.3% (13/16) had high level resistance with MIC ≥32mg/L. The 53 RIF-resistant strains had a wide MIC distribution: 1.5–4mg/L (*n* = 32), 6–16mg/L (*n* = 9), 24mg/L (*n* = 1), and ≥32mg/L (*n* = 11). As for the MIC values against AML, more than half of the 87 strains had a MIC ≤0.016mg/L (*n* = 49), while 29 strains had a MIC in the range of 0.023–0.125mg/L, eight had a MIC value at 0.19mg/L, and the remaining one strain had a MIC value at 0.25mg/L. All the 87 strains were susceptible to TET and FZD. The MIC values against TET were ≤0.125mg/L in more than 90% of the strains (79/87), and nearly half of the strains tested against FZD had a MIC ≤0.125mg/L (*n* = 41).

## Discussion

*Helicobacter pylori* infection is a bacterial infectious disease and therefore successful *H. pylori* eradication is mainly dependent on the choice of susceptible antibiotics. However, since antimicrobial susceptibility testing for *H. pylori* is almost universally unavailable, it has been recommended that the choice of an effective empirical eradication therapy is based on region and population-specific antibiotic resistance patterns. With 87 *H. pylori* pediatric strains isolated in Southwest China from three different ethnic groups (Han, Tibetan, and Yi), we demonstrated a very high overall resistance rate to CLR (55.2%), MTZ (71.3%), and RIF (60.9%). The overall resistance rate to LEV was 18.4%. None of these pediatric strains were resistant to AML, TET, and FZD.

To our knowledge, this is the first study to report the antibiotic susceptibility profile among pediatric patients in Southwest China. The primary CLR resistance among the 53 treatment-naïve pediatric patients was 45.3% in this study, which was much higher than the primary CLR resistance rates reported among pediatric patients in Southeast China including Zhejiang province (16.4%; Li et al., 2017) and Jiangxi province (22%; Liu et al., 2019). Interestingly, the primary CLR resistance rate among pediatric patients in Beijing (northern China) was reported as high as 84.9% in 2011 (Liu et al., 2011). In view of the extremely high primary and secondary CLR resistance, the CLR-based regimens (even with the addition of bismuth) are no longer suitable as empiric treatment therapies for pediatric patients in Southwest China. However, of note is that if CLR is known to be susceptible by antimicrobial susceptibility testing, this antibiotic can be confidently prescribed to achieve successful *H. pylori* eradication (Jones et al., 2017; Tang et al., 2020a).

The recorded primary MTZ resistance among the pediatric population examined was 73.6%, which was much higher than the primary MTZ resistance reported among pediatric patients in Jiangxi province (46%; Liu et al., 2019), but it is comparable to the regional MTZ resistance rate recently reported among pediatric patients in Zhejiang province (75.2%; Li et al., 2017). Simultaneous resistance to CLR and MTZ was present in 28.3% of the 53 treatment-naïve patients, and in 52.9% of the 34 pediatric patients with previous treatment in this study, suggesting that the concomitant regimen (PPI, AML, CLR, and MTZ) might not be suitable as empiric therapy for these pediatric patients. For *H. pylori* eradication in adults, the MTZ resistance can be well overcome by increasing the dosage of MTZ or the dosage of AML (Liang et al., 2013; Zhang et al., 2015; Chen et al., 2019). Whether increasing the dosage of MTZ or AML can overcome MTZ resistance has not been studied in pediatric patients. Interestingly, it has been reported that among pediatric patients in Japan with MTZ resistance surpassing 20% (Okuda et al., 2017), the standard triple therapy with the combination of PPI, AML, and MTZ can still achieve an excellent eradication rate of more than 95% (Okuda et al., 2017; Mabe et al., 2018). This suggests that low level MTZ resistance may exert very limited influence on *H. pylori* eradication. However, considering the high MTZ resistance rate observed in this study, the bismuth quadruple therapy through the addition of bismuth to the standard triple therapy (PPI, AML, and MTZ) would be suitable as first-line empiric treatment regimen for pediatric patients in China. The major role of bismuth is to increase an additional 30–40% eradication success for resistant *H. pylori* infections. Of note is that bismuth in China has only been recommended for patients older than 6years (Xu et al., 2015).

The use of quinolones is contraindicated in patients younger than 18years in China. However, the primary LEV resistance rate among the pediatric patients in this study was 15.1%, which seemed higher than the primary LEV resistance rate reported in Zhejiang province (6.7%), but it is comparable to the primary LEV resistance reported in Beijing (13.7%). As *H. pylori* infection is usually acquired in childhood mainly through intra-family transmission, the high LEV resistance in children might be explained by the transmission of LEV-resistant strains from parents to children.

RIB has been recommended for refractory *H. pylori* treatment (Gisbert and Calvet, 2012; Fallone et al., 2016; Chey et al., 2017; Malfertheiner et al., 2017), while in the laboratory, the MICs of RIF are used for screening RIB resistance (Hays et al., 2018). In this study, both primary and secondary RIF resistance rates surpassed 60%. Of the 53 RIF-resistant strains, 11 strains exhibited high level resistance to RIF with MIC values being recorded ≥32mg/L (Figure 1). The extremely high RIF resistance might be partly ascribed to the frequent use of RIF/RIB for the treatment of *Mycobacterium tuberculosis*, which remains a serious public health problem in Southwest China.

Resistance to AML was not detected among the pediatric patients in this study, which was in line with the negligible AML resistance rate (0.06%) reported among pediatric patients in Zhejiang province (Li et al., 2017), and the nil AML resistance among pediatric patients in Beijing (Liu et al., 2011). AML resistance in children has also been reported as nil or negligible in many other countries, including Portugal (0.0%; Silva et al., 2018), Spain (0.0%; Montes et al., 2015), Germany (0.8%; Regnath et al., 2017), Italy (0.0%; Manfredi et al., 2015), and Poland (0.0%; Oleastro et al., 2011). As none of strains isolated from the treatment-experienced pediatric patients were found to be resistant to AML, it is not necessary to consider AML resistance in patients with previous treatment failures. Nil resistance to TET and FZD was also observed among the pediatric patients in this study. However, due to the side effects of teeth discoloration and enamel hypoplasia, TET is contraindicated in children younger than 8years. The use of FZD is only indicated for the treatment of refractory *H. pylori* infections. Of note, FZD is forbidden for use in pediatric patients younger than 14years.

Our study has several limitations. First, we only used the classical phenotypic antimicrobial susceptibility testing method to assess the antibiotic resistance profile in this study. Genotypic molecular tests need to be considered in future studies for the detection of CLA and LEV resistance. Second, our study was conducted among pediatric patients in a single tertiary hospital, and so our findings may not be applied in the general pediatric patients in Southwest China. Third, the number of pediatric patients included in this study was relatively small, which might be the reason of not being able to detect the difference in antibiotic resistance rate among different ethnic groups. Future studies are needed to include more pediatric patients to investigate factors (e.g., age, race, gender, and prior treatment history) that are likely to influence antibiotic resistance profiles in pediatric *H. pylori* strains.

In summary, we demonstrated very high antibiotic resistance rates to CLR, MTZ, LEV, and RIF in this study, whereas resistance to AML, TET, and FZD was not detected. Our data suggest that the standard CLR-based triple therapy should be abandoned as empiric therapy for pediatric patients in Southwest China. AML should be an indispensable antibiotic component of the initial or rescue *H. pylori* eradication regimens provided that the pediatric patients are not allergic to AML. Considering the extremely high CLR and MTZ resistance, and the limited choice of antibiotics for *H. pylori* treatment in children, TET and FZD may be considered for treating refractory *H. pylori* infections in pediatric patients older than 8- and 14-years.

## Data Availability Statement

The original contributions presented in the study are included in the article/supplementary material, further inquiries can be directed to the corresponding authors.

## Ethics Statement

The studies involving human participants were reviewed and approved by Ethics Committee of West China Second Hospital of Sichuan University. Written informed consent to participate in this study was provided by the participants’ legal guardian/next of kin.

## Author Contributions

HL worked on design and concept of this work, drafting, and revising the manuscript. JL, JD, and ZW implemented this study and analysis of data. CW provided the funding support and project administration. All authors contributed to the article and approved the submitted version.

### Conflict of Interest

The authors declare that the research was conducted in the absence of any commercial or financial relationships that could be construed as a potential conflict of interest.

## Footnotes

^1^https://www.dayi.org.cn/
